# Pattern of SQSTM1 Gene Variants in a Hungarian Cohort of Paget’s Disease of Bone

**DOI:** 10.1007/s00223-020-00758-4

**Published:** 2020-09-25

**Authors:** Judit Donáth, Bernadett Balla, Márton Pálinkás, Rita Rásonyi, Gyula Vastag, Nerea Alonso, Beatriz Larraz Prieto, Mahéva Vallet, Stuart H. Ralston, Gyula Poór

**Affiliations:** 1 National Institute of Rheumatology and Physiotherapy, Budapest, Hungary; 2 PentaCore Laboratories, Budapest, Hungary; 3 Corvinus University of Budapest, Budapest, Hungary; 4 Rheumatology and Bone Disease Unit, Centre for Genomics and Experimental Medicine, IGMM, University of Edinburgh, Edinburgh, UK; 5 Semmelweis University, Budapest, Hungary

**Keywords:** Paget’s disease of bone, *SQSTM1* genetic variants, Sequencing, Hungarian origin

## Abstract

Paget’s disease of bone (PDB) is characterized by focal or multifocal increase in bone turnover. One of the most well-established candidate genes for susceptibility to PDB is Sequestosome 1 (*SQSTM1*). Mutations in *SQSTM1* have been documented among Western-European, British and American patients with PDB. However, there is no information on *SQSTM1* mutation status in PDB patients from the Central- and Eastern-European regions. In this study, we conducted a mutation screening for *SQSTM1* gene variants in 82 PDB patients and 100 control participants in Hungary. Mutations of *SQSTM1* were detected in 18 PDB patients (21.95%); associations between genotype and clinical characteristics were also analyzed. Altogether, six different exonic alterations, including two types of UTR variants in the *SQSTM1* gene, were observed in our PDB patients. Similarly, to previous genetic studies on Paget’s disease, our most commonly detected variant was the c.1175C > T (p.Pro392Leu) in nine cases (four in monostotic and five in polyostotic form). We have surveyed the germline *SQSTM1* variant distribution among Hungarian patients with PDB. We also highlighted that the pattern of the analyzed disease-associated pathophysiological parameters could partially discriminate PDB patients with normal or mutant *SQSTM1* genotype. However, our findings also underline and strengthen that not solely *SQSTM1* stands in the background of the complex PDB etiology.

**Electronic supplementary material:**

The online version of this article (10.1007/s00223-020-00758-4) contains supplementary material, which is available to authorized users.

## Introduction

Paget’s disease of bone (PDB) is characterized by focal areas of rapid bone remodeling. This remodeling imbalance leads to mechanically weak, disorganized bone structure with irregular collagen fibers [1–3]. The pathological changes may result in mild to severe clinical manifestations in that some patients have no symptoms whereas others experience bone pain, deformity, pathological fractures, bone deformity and deafness [4–6]. There are marked differences in prevalence in different geographical regions [7, 8]. There has been a decline in both prevalence and severity of the disease in many but not all regions in the last twenty years [9, 10].

The etiology of the disease is incompletely understood but genetic factors play an important role. The most important susceptibility gene is Sequestosome 1 (*SQSTM1*) but genome-wide association studies have identified several additional loci that predispose to classical PDB at a genome wide significant level [11–15].

Population-based distribution of alterations is well documented in more details among Western-European, British, American and Australian origins as well [14, 16]. However, there are no literature data in relation to the mutational pattern of PDB patients in the Central- and Eastern-European region.

This study describes the *SQSTM1* mutations and the clinical parameters of Hungarian PDB patients.

## Materials and Methods

### Participants and Routine Methods

The study participants were treated between 2006–2016 at the National Institute of Rheumatology and Physiotherapy, Budapest, Hungary. Clinical diagnosis was made on whole body scan to all PDB patients, radiographs of all bones displaying an uptake in bone scan to perform differential diagnosis to all. The number of bones affected by PDB was established from 99Tc-labelled methylene diphosphate (MDP)-labelled bone scintigraphy. We measured routine laboratory parameters in all patients and in the case of high GGT, abdominal ultrasound was also made [19, 20]. Plasma total alkaline phosphatase (ALP) activity was measured using a Dimension Xpand analyzer (Siemens Diagnostics, Germany), with a reference range of 98–280 U/L. ALP at the time of diagnosis was used in the analysis.

The presence of bone deformity was recorded by the attending physicians who were asked to assess whether the patient had clinical evidence of bone deformity or not on physical examination [17, 18]

We excluded people with rare PDB-like syndromes such as familial expansile osteolysis, expansile skeletal hyperphosphatasia and juvenile Paget’s disease of bone. In the study only untreated PDB cases at the time of diagnosis were included. Genetic analysis was performed on 82 Hungarian patients (59% male, 41% female) with Paget’s disease of bone. The majority of PDB patients were unrelated (78 of 82), four patients (two pairs of siblings) had family history. Age at recruitment ranged from 45 to 94 years with an average (± SD) of 71.6 ± 8.74 years (Table 1).

**Table 1:** Clinical characteristics of PDB patients according to *SQSTM1* mutation status

Variable	*SQSTM1* mutation: yes (*n* = 18)	*SQSTM1* mutation: no (*n* = 64)	*p*-value
Age at first diagnosis (year)	59.4 ± 10.07	62.2 ± 9.85	0.15
Age at the time of the study	70.11 ± 6.23	72.05 ± 9.32	0.15
Biological sex, number (%) of males	9 (50.0%)	39 (60.9%)	0.40
Family history, number (%) of yes	3 (16.7%)	1 (1.56%)	0.0075
Alkaline phosphatase at diagnosis (ALP)*	787 ± 535.5	822 ± 820.1	0.4158
Other pain (osteoarthritis), number (%)	6 (33.3%)	29 (45.31)	0.42
Bone pain thought to be caused by PDB (%)	3 (16.7%)	0 (0.0%)	0.009
Polyostotic, *n* (%)	6 (33.3%)	16 (25.0%)	0.55
Number of bones affected	1.55 ± 0.92	1.37 ± 0.75	0.22
Deformity number due to PBD (*n*)	0.28 ± 0.57	0.17 ± 0.38	0.76
Fracture of affected, number (%)	0 (0.0%)	1 (1.56%)	1.00
Surgery due to PDB, *n* (%)	2 (11.1%)	3 (4.7%)	0.30
Skull disease, *n* (%)	2 (11.1%)	9 (14.06%)	1.00

For each continuous variable, the mean ± SD are shown and for categorical variables the number of occurrences and their relative frequencies are shown. The *p*-values refer to differences between groups assessed by Students *t*-test for continuous variables or Fisher’s exact test for categorical variables

*Reference range for ALP: 98–280 U/L

We collected 100 control participants from our centre, who were affected by mild degenerative rheumatological conditions as spondylosis, scoliosis or osteoarthritis. The controls came from the same geographic area as the PDB patients. They had the same ethnicity, they were not related, and were not age-matched to the PDB patients. PDB was ruled out in controls by routine laboratory parameters (plasma alkaline phosphatase and GGT level measuring). The control patients did not have evidence of bone pain and their ALP levels were in the normal range, but scintigraphy was not routinely performed in these individuals. The average (± SD) age of the control group was 62.2 ± 11.91 years, with 44% male and 56% female participants.

### DNA Isolation and Sequencing

Genomic DNA was isolated from EDTA-anticoagulated peripheral blood samples using QIAamp DNA Blood Mini Kit on automated QIAcube HT System (QIAGEN, Hilden, Germany) according to the manufacturer’s instructions. We assessed the quality and concentration of the isolated DNA using NanoDrop spectrophotometer (NanoDrop Technologies, Montchanin, DE, USA) at 260/280 nm.

For variant screening, exons 7 and 8 were targeted, including the intron–exon boundaries. The primer set used was previously designed by Hocking LJ and coworkers [19, 20]: For exon 7: forward 5′TTAAAGTCACGCTGGGAACCTGCT3′; reverse‐5′AGGGCAGGATGCTCTAAAGGG3′. For exon 8: forward‐5′TCTGGGCAGGCTCGGACACT3′; reverse‐5′CCCTAAATGGCTTCTTGCACCC3′. We administered 35 cycles for PCR amplification on 65 °C annealing temperature with Qiagen Taq polymerase based on the manufacturer's suggestions in DNA Engine Tetrad® 2 Thermal Cycler machine (BioRad, Hercules, CA, USA). Mutations were confirmed on two independent PCR products. Enzymatic PCR products cleaning with ExoSAP-IT (Affymetrix, Santa Clara, CA, USA) as well as Sanger sequencing were performed at IGMM sequencing services (University of Edinburgh, MRC Institute of Genetics and Molecular Medicine). Sequencing data were analyzed using Mutation Surveyor v3.30 software. Variant classification has been befallen on the current ACMG standards and guidelines 2015 [21]. PDB patients were defined as ‘mutant’ if any known pathogenic or uncertain variants were detected in the analyzed exonic regions. We utilized a clinically certified annotation platform VarSome to interpret our sequence data and to summarize the possible impact of the described variants [22].

Due to our sequencing design, we could also identify intronic variants in the gene. We have used ESEfinder 2.0 software (https://krainer01.cshl.edu/tools/ESE2/index.html) to predict in silico whether the intronic variants could show a pathogenic behaviour. This software identifies the intronic binding sites for exonic splicing enhancers (ESE).

### Statistical Analysis

Student’s *t* test was used to evaluate differences between patients with and without *SQSTM1* mutations for continuous variables and Pearson’s chi-square test was used for categorical variables. In cases where chi-square was suspect (20% of the cells had expected count less than 5), Fisher’s exact test (two-sided) was also used.

## Results

The clinical characteristics of patients are summarized in Table 1. Patients with *SQSTM1* mutations differed significantly from those without mutations in terms of family history and bone pain thought to be caused by PDB. No significant differences were detected between genotype groups in term of other parameters

Genetic analysis was completed in all the examined 182 patients. Six different exonic alterations including two types of UTR variants in the gene were observed in PDB patients (listed in Table 2). Eighteen out of the 82 PDB-affected participants (21.9%) carried a mutation. Four patients (two pairs of siblings) had family history. One of the two brothers (aged 71) carried the variant c.*174insTG; the other (69) did not show any pathogenic *SQSTM1* gene variations. Both, the older (74) female sibling and her younger (70) brother, carried the NM_003900.5:c.1175C > T, p.Pro392Leu mutation. Phenocopy refers to a non-hereditary change, in this case due to the absence of the mutation in one of the siblings. This might be a possible scenario; however, no relevant clinical differences were found.

**Table 2:** Descriptive of the identified variants in PDB and control patients

dbSNP	Coding variant NM_003900.5:	Protein effect	Variant type	ACMG classification	MAF	Incidence PDB/control
rs776749939	c.1160C > T	p.Pro387Leu	Missense	Likely pathogenic	*T* = 0.00004	1/0
rs104893941	c.1175C > T	p.Pro392Leu	Missense	Pathogenic/likely pathogenic	*T* = 0.0009	9/1
rs1254158201	c.1185dup	p.Glu396Ter	Nonsense	Pathogenic	–	1/0
rs143511494	c.1231G > A	p.Gly411Ser	Missense	Likely pathogenic/VUS	A = 0.00005	1/0
rs10688915	c.*174_*175 =	–	3′ UTR	likely benign	TG = 0.0557	6/0
rs765964997	c.*2_*4delCAC	–	3′ UTR	VUS	AC = 0.0000119	1/0
rs155790	c.*83G =	–	3′ UTR	VUS	A = 0.0053	0/1

The HGVS (Human Genome Variation Society) recommendations were used for the nomenclature of *SQSTM1* sequence variants

Sequence variants were classified according to The American College of Medical Genetics and Genomics (ACMG) criteria. VUS—Variant of Uncertain Significance

*MAF* Minor allele frequency

Similarly, to previous genetic studies on PDB, the most commonly detected variant was NM_003900.5:c.1175C > T, p.Pro392Leu (rs104893941), found in nine cases (four in monostotic and five in polyostotic form). We identified an additional known nonsense variation NM_003900.5:c.1185dup, p.Glu396Ter (rs1254158201) and two missense alterations NM_003900.5:c.1160C > T, p.Pro387Leu (rs776749939) and NM_003900.5:c.1231G > A, p.Gly411Ser (rs143511494). We described a 3′ UTR variant, namely NM_003900.5:c.*174_*175 = (rs10688915) in six cases (five in monostotic and one in polyostotic form). Furthermore, we found a 3-bp deletion NM_003900.5:c.*2_4*delCAC (rs765964997) located in the 3′ UTR region of exon 8. This deletion was carried by a man with monostotic phenotype who was 67 years old at the time of diagnosis. There was not a clear difference in clinical characteristics of patients who carried different *SQSTM1* mutations although interpretation of these data was made difficult by the small numbers. Characteristics of patients with different mutations is shown in Supplementary Table S1.

According to the description of variants in control individuals, we found NM_003900.5:c.*83 = in 3′ UTR of exon 8 (rs155790; MAF: A = 0.0053; ACMG criteria to sequence variant classification: VUS—variant of uncertain significance) in a 57-year-old man and the NM_003900.5:c.1175C > T1175C›T, p.Pro392Leu (rs104893941) in 78-year old female control participant. This individual had no signs of PDB or family history of the disease.

To gain high quality coverage of the regions of interest, the primers flanked some parts of the nearby introns. In all three intronic variants were recognized in the entire sample group. These were the following: NM_003900.5:c.970-59 T > C (rs155788; MAF: C = 0.19), NM_003900.5:c.970-93G > A (rs155787; MAF: G = 0.35) and NM_003900.5:c.970-109C > G (rs2241350; MAF: G = 0.15).

The incidence of the intronic variants are listed below: c.970-59 T > C: Cases: 14/82 *vs.* Controls: 22/100; c.970-93G > A: Cases: 62/82 *vs.* Controls: 75/100; c.970-109C > G: Cases: 18/82 *vs.* Controls: 16/100, respectively.

We have found that rs155788 variant creates a new potential binding site for SF2/ASF (threshold 2.56) and SR35 proteins (threshold 3.66). The rs155787 showed no changes in the binding sites for exonic splicing enhancers and rs2241350 variants alters a binding site for SRp40 protein (threshold 2.97).

## Discussion

This is the first study about the distribution of germline mutations among Hungarian patients diagnosed with PDB. Population-based screening of variants has not yet been reported from the Central European region and especially in any Hungarian PDB cohort. To map the detailed genetic aspects of a hereditary disease, it is indispensable to have a wide range of genomic DNA data from different nationalities and ethnic groups.

Eighteen out of the 82 PDB-affected participants (23.2%) harbored a mutation in *SQSTM1* gene. The incidence of the p.Pro392Leu mutations in PDB varies in different populations worldwide from 2.4–16%. Currently, there are 139 annotated variants from which 34 were classified as pathogenic in the ClinVar database. Based on recent literature about PDB, most of the clinically relevant causal sequence variants are clustered within the ubiquitin-associated (UBA) domain encoded by exon 7–8 [12, 14].

The distribution by country are as follows: 2.4–3.7% and 5.4–7.3% in Dutch and Belgian cohort, respectively, 4.8–5.4% in Italian series and 15.6% in Spanish PDB participants as well as 8.6–8.9% in those of British ancestry and 9–16% in the French-Canadian population.[12, 14, 15, 23]. Consistent with other studies, p.Pro392Leu (rs104893941, c.1175C > T) was the most frequently identified pathogenic single-nucleotide variant (SNV) in our patients. The substitution of Pro to Leu disrupts the ubiquitin-binding activity of Sequestosome 1. Nearly, half (47%0.37%) of the detected alterations was this recurrent missense variant as well as 11% (9/82) of our cases carrying the p.Pro392Leu mutation. Among the noticed SNVs, one nonsense NM_003900.5:c.1185dup (p.Glu396Ter) variant—causing premature termination—was revealed in a man, who was diagnosed with monostotic PDB affecting the scapula at age 50 years. This variant effectively removes the large majority of UBA domain in the gene product [24].

Furthermore, we determined two distinct 3′ UTR variants which cause a 2-bp TG insertion (NM_003900.5:c.*174_*175 =) or 3-bp deletion (NM_003900.5:c.*2_*4delCAC) after the stop codon and they were classified as likely benign or VUS (Variant of Uncertain Significance), respectively. In one genetic study of Lucas et al., 3′ UTR polymorphisms have been noted without marked differences in their allele frequencies between British PDB cases and controls [25]. It has been well-documented that microRNAs (miRs) widely modulate gene expression through binding the 3′ untranslated region of target mRNAs [26]. Although not yet proven today, it cannot be ruled out, so the two 3′UTR variants we found may affect miRNA binding sites and regulate *SQSTM1* expression.

From our 100 control participants, two showed *SQSTM1* variants. One carried a NM_003900.5:c.*83 = (G > A substitution) variant—with clinically uncertain importance—in 3′ UTR of exon 8, and one exhibited the pathogenic c.1175C > T (p.Pro392Leu) alteration. According to the original article by Visconti MR et al., the overall frequency of alterations is on average 0.07% among unaffected controls [27] and, consistently, the allele frequency of p.Pro392Leu in the EXaC database has been reported to be 0.10%. In this regard it is interesting to note that some patients with pathogenic variants in *SQSTM1* do not develop PDB but instead present with neurological disease such as amyotrophic lateral sclerosis (ALS) as part of multisystem proteinopathy syndromes [28] The reason underlying these different presentations remains incompletely understood but there is evidence that digenic inheritance of more than one variant may play a role [29].

There is compelling evidence that intronic variants have been potentially associated to the pathogenesis of ALS and early-onset Alzheimer dementia, as well [30–32]. However, clinical significance of the intronic mutations in PDB has not yet been proven.

To summarize the findings of genetic analysis and the clinical characteristics of Hungarian PDB cohort, we can conclude that the alterations in our PDB patients (21.95%) lay on the higher end of the range (10%-21%) reported in the literature. The recurrent c.1175C > T (p.Pro392Leu) sequence variant incidence has been correlated with the globally available average allele frequency (11% *vs.* 7.1%) among unrelated PDB cases. Likewise, we have seen some minor variations in the clinical manifestation and disease phenotypes. Family history of PBD and bone pain thought to be caused by PBD occurred significantly higher in case of *SQSTM1* mutations. Although *SQSTM1* has been associated with severity [27], previous studies have not indicated more prevalence of Pagetic pain. These few differences can be explained by the different demographic features and the relatively low sample size.

We have surveyed the germline variant distribution among Hungarian patients with Paget’s disease of bone. We also highlighted that the pattern of the analyzed disease-associated pathophysiological parameters could partially discriminate PDB patients with normal or mutant genotype. The suspected patterns need to be verified and tested on a larger sample; the next step of this investigation.

## Electronic supplementary material

Below is the link to the electronic supplementary material.

Supplementary file1 (DOCX 13 kb)
