# Supplementary material for: Pattern of SQSTM1 Gene Variants in a Hungarian Cohort of Paget’s Disease of Bone
Source: Calcif Tissue Int. 2020 Sep 25;108(2):159–64. doi: 10.1007/s00223-020-00758-4 (PMC7819901; doi:10.1007/s00223-020-00758-4)
Supplement: Supplementary file 1 — Supplementary file1 (DOCX 13 kb) [file 223_2020_758_MOESM1_ESM.docx]

Supplementary Table S1

Clinical characteristics of PDB patients relative to the six SQSTM1 mutations

|  | **rs104893941**  **c.1175C>T**  **(n=9)** | **rs1254158201**  **c.1185insT**  **(n=1)** | **rs143511494**  **c.1231G>A**  **(n=1)** | **rs10688915**  **c.*174_*175insTG**  **(n=6)** | **rs765964997**  **c.*2_*4delCAC**  **(n=1)** |
| --- | --- | --- | --- | --- | --- |
| Age at first diagnosis (year) | 61±10.6 | 50 | 59 | 57±11.2 | 67 |
| Age at the time of the study | 71±5.6 | 60 | 68 | 69±6.5 | 78 |
| Biological sex, number (%) of males | 5 (55.6) | 1 (100.0) | 0 (100.0) | 1 (16.7) | 1 (100.0) |
| Family history, number (%) of yes | 2 (22.2) | 0 (0.0) | 0 (0.0) | 1 (16.7) | 0 (0.0) |
| Alkaline phosphatase at diagnosis (ALP)^*^ | 943±665.8 | 602 | 623 | 508±202.4 | 1,400 |
| Bone pain, number (%) | 2 (22.2) | 1 (100.0) | 0 (0.0) | 2 (33.3) | 1 (100.0) |
| Bone pain thought to be caused by PDB (%) | 2 (22.2) | 0 (0.0) | 0 (0.0) | 1 (16.7) | 0 (0.0) |
| Polyostotic, n (%) | 5 (55.6) | 0 (0.0) | 0 (0.0) | 1 (16.7) | No (0.0) |
| Number of bones affected | 2±1.12 | 1 | 1 | 1.2±0.41 | 1 |
| Deformity number due to PBD (n) | 0.55±0.73 | 0 | 0 | 0 | 0 |
| Fracture of affected bones, number (%) | 0 (0.0) | 0 (0.0) | 0 (0.0) | 0 (0.0) | 0 (0.0) |
| Surgery due to PDB, n (%) | 2 (22.2) | 0 (0.0) | 0 (0.0) | 0 (0.0) | 0 (0.0) |
| Skull disease, n (%) | 2 (0.22) | 0 (0.0) | 0 (0.0) | 0 (0.0) | No (0.0) |
